# Reduced IL-10 Production in Fetal Type II Epithelial Cells Exposed to Mechanical Stretch Is Mediated via Activation of IL-6-SOCS3 Signaling Pathway

**DOI:** 10.1371/journal.pone.0059598

**Published:** 2013-03-19

**Authors:** Michael A. Hokenson, Yulian Wang, Renda L. Hawwa, Zheping Huang, Surendra Sharma, Juan Sanchez-Esteban

**Affiliations:** Department of Pediatrics, Women & Infants Hospital of Rhode Island and The Warren Alpert Medical School of Brown University, Providence, Rhode Island, United States of America; University of Alabama-Birmingham, United States of America

## Abstract

An imbalance between pro-inflammatory and anti-inflammatory cytokines is a key factor in the lung injury of premature infants exposed to mechanical ventilation. Previous studies have shown that lung cells exposed to stretch produces reduced amounts of the anti-inflammatory cytokine IL-10. The objective of these studies was to analyze the signaling mechanisms responsible for the decreased IL-10 production in fetal type II cells exposed to mechanical stretch. Fetal mouse type II epithelial cells isolated at embryonic day 18 were exposed to 20% stretch to simulate lung injury. We show that IL-10 receptor gene expression increased with gestational age. Mechanical stretch decreased not only IL-10 receptor gene expression but also IL-10 secretion. In contrast, mechanical stretch increased release of IL-6. We then investigated IL-10 signaling pathway-associated proteins and found that in wild-type cells, mechanical stretch decreased activation of JAK1 and TYK2 and increased STAT3 and SOCS3 activation. However, opposite effects were found in cells isolated from IL-10 knockout mice. Reduction in IL-6 secretion by stretch was observed in cells isolated from IL-10 null mice. To support the idea that stretch-induced SOCS3 expression via IL-6 leads to reduced IL-10 expression, siRNA-mediated inhibition of SOCS3 restored IL-10 secretion in cells exposed to stretch and decreased IL-6 secretion. Taken together, these studies suggest that the inhibitory effect of mechanical stretch on IL-10 secretion is mediated via activation of IL-6-STAT3-SOCS3 signaling pathway. SOCS3 could be a therapeutic target to increase IL-10 production in lung cells exposed to mechanical injury.

## Introduction

Bronchopulmonary dysplasia (BPD) remains the most common cause of lung disease in infancy and is a major cause of neonatal morbidity and mortality [1]. It is estimated that 25% of infants with a birth weight of less than 1500 grams have BPD [2]. Although it is well accepted that the etiology of BPD is multi-factorial, injury secondary to mechanical ventilation plays a central role [3].

Tracheal aspirates from infants with BPD have shown elevated concentrations of pro-inflammatory cytokines, such as IL-6 and IL-8, and decreased concentrations of the anti-inflammatory cytokine IL-10 [4]. In addition, IL-10 expression in the placenta has been associated with a decreased risk of developing BPD [5]. These investigations support the concept that an imbalance between pro-inflammatory and anti-inflammatory cytokines may play a critical role in the pathogenesis of BPD. Previous studies of BPD have focused on inflammatory cells such as neutrophils and macrophages and their ability to initiate an inflammatory response [6]. However, the role of type II epithelial cells in the development of BPD is not well established. Previous observations from our laboratory have shown that fetal type II epithelial cells exposed to mechanical stretch have decreased IL-10 secretion as compared to controls [7]. However, the mechanisms behind the decreased production of IL-10 by type II epithelial cells following mechanical stretch remain poorly understood.

The IL-10 pathway begins with the receptor complex, which is a tetramer consisting of two subunits (IL-10 R1 and IL-10 R2). The binding of IL-10 to the extracellular domain of IL-10 R1 activates the receptor associated kinases JAK-1 and TYK2, which then phosphorylate specific tyrosine residues on the intracellular domain of the IL-10 R1 chain [8]. Once phosphorylated, the tyrosine residues serve as docking sites for the transcription factor STAT3. Upon activation, STAT3 homodimerizes and translocates to the nucleus where it binds with IL-10 responsive genes [9], [10].

One of the IL-10 responsive genes is SOCS3 (suppressor of cytokine signaling 3) [10]. SOCS3 plays an important role in negative regulation of inflammatory response. SOCS3 attenuates signaling by blocking JAK tyrosine kinase activity or STAT activation [11]. The IL-10-mediated induction of SOCS3 in macrophages has led to the notion that SOCS3 is an essential component of the anti-inflammatory effect mediated by IL-10 [12]. SOCS3 also serves as a negative feedback regulator of pro-inflammatory cytokines, such as IL-6 and IL-8 [13].

The objective of these studies was to analyze the cell signaling mechanisms responsible for the decreased IL-10 production in type II cells exposed to exaggerated mechanical stretch. We hypothesized that this process is mediated via inhibition of IL-10 signaling pathway. We speculated that the increased production of pro-inflammatory cytokines seen in type II cells exposed to mechanical stretch would induce SOCS3 expression, and in turn decrease IL-10 production given the dual role of this protein as a negative feedback regulator of both pro-inflammatory and anti-inflammatory cytokines.

## Materials and Methods

### Cell Isolation and Stretch Protocol

This study was carried out in strict accordance with the recommendations in the Guide for the Care and Use of Laboratory Animals of the National Institutes of Health. The protocol was approved by the Lifespan Institutional Animal Care and Use Committee, Providence, RI (Protocol # 0195-09). Fetal mouse lungs were obtained from timed-pregnant C57BL6 wild-type and IL-10 knockout mice at embryonic days 18 (saccular stage of lung development) and type II cells were isolated as previously described [14]. Briefly, after collagenase or dispase digestion, cell suspensions were sequentially filtered through 100-, 30-, and 20-µm nylon meshes using screen cups (Sigma). Clumped nonfiltered cells from the 30- and 20-µm nylon meshes were collected after several washes with DMEM to facilitate the filtration of nonepithelial cells. Further type II cell purification was achieved by incubating the cells in 75-cm^2^ flasks for 30 min. Non-adherent cells were collected and cultured overnight in 75-cm^2^ flasks containing serum-free DMEM. After overnight culture, cells were harvested with 0.25% (wt/vol) trypsin in 0.4 mM EDTA, and plated (around 50% confluency) on Bioflex multiwell plates (Flexcell International, Hillsborough, NC) precoated with fibronectin [1.5 µg/cm^2^]. Monolayers were maintained in culture for 1–2 days until they were approximately 80% confluents and then were mounted in a Flexcell FX-4000 Strain Unit (Flexcell International). Equibiaxial cyclical strain regimen of 20% was applied at intervals of 40 cycles/min for different lengths of time. This regimen, which roughly corresponds to a lung inflation of 80% of total lung capacity in adult rats [15], was chosen to mimic lung cells injury. Cells were grown on nonstretched membranes in parallel and were treated in an identical manner to serve as controls.

### Concentrations of IL-10 and IL-6 in the Supernatant

After experiments, the cell culture medium was collected, centrifugated to remove cell debris and stored at −80°C before analysis. Cell monolayers from the BioFlex plates were lysed with ice-cold RIPA buffer (150 mM NaCl, 100 mM Tris base, pH 7.5, 1% deoxycholate, 0.1% SDS, 1% Triton X-100, 3.5 mM Na_3_VO_4_, 2 mM PMSF, 50 mM NaF, 100 mM sodium pyrophosphate) with protease inhibitors (10 µg/ml leupeptin, 10 µg/ml aprotinin, 143.5 µM aminoethyl benzenesulfonyl fluoride). Lysates were centrifuged and total protein content was determined by the bicinchoninic acid method. IL-10 and IL-6 concentrations in the supernatant were measured using commercial ELISA kits (IL-10: Quantikine, R&D Systems, Minneapolis, MN, cat # M1000; IL-6 EIA kit, Enzo Life Sciences, Farmingdale, NY, cat # ADI-900-045) according to the manufacture’s recommendations. The optical density was determined photometrically at 450 nm using the ELISA plate reader EL_x_800 (Bio-Tek Instruments). Results were normalized to the cell lysate concentration in each sample as a representation of the number of cells added to the wells.

### Real-time PCR (qRT-PCR)

Total RNA was isolated as previously described [16] and purified further using the Turbo DNA-free kit (Ambion). One microgram of total RNA was reverse-transcribed into cDNA using the iScript™ cDNA Synthesis Kit (Bio-Rad) according to the manufacturer’s instructions. Pre-designed TaqMan® primers were purchased from Assays-on-Demand™ Gene Expression Products (Applied Biosystems).

The following primers were used: IL-10R1 (cat #: Mm00434151_m1), IL-10R2 (cat #: Mm00434157_m1) and IL-10 (cat #: Mm00439614_m1) (Applied Biosystems). To amplify the cDNA by qRT-PCR, 4 µl of the resulting cDNA were added to a mixture of 10 µl of TaqMan Gene Expression Master Mix (Applied Biosystems) and Assays-on-Demand™ Gene Expression Assay Mix containing forward and reverse primers and TaqMan labeled probe (Applied Biosystems). Standard curves were generated for each primer set and housekeeping gene GAPDH (cat #: Rn99999916_m1). Linear regression revealed efficiencies between 96 and 99%. Therefore, fold expressions of stretched samples relative to controls were calculated using the ΔΔC_T_ method for relative quantification (RQ) as previously described [17]. Samples were normalized to GAPDH. The reactions were performed in a 7500 Fast Real-Time PCR System (Applied Biosystems) with the following parameters: 50°C –2 min, 95°C –10 min, and 45 cycles of 95°C –15 s, 60°C –1 min. All assays were performed in triplicate.

### Western Blot Analysis

Monolayers were lysed with RIPA buffer containing protease inhibitors. Lysates were centrifuged and total protein contents were determined by the bicinchoninic acid method. Equal amount of protein lysate samples (20 µg) were fractionated by NU-PAGE Bis-Tris (4–12%) gel electrophoresis (Novex, San Diego, CA) and transferred to polyvinylidene difluoride membranes. Blots were hybridized with phospho-specific antibodies to JAK1 (cat # 3331, Cell Signaling Technology, Beverly, MA) TYK2 (cat #: sc-169, Santa Cruz, CA) and STAT3 (cat #: 9134S, Cell Signaling Technology, Beverly, MA). Blots were also hybridized to total SOCS3 (cat #: sc-9023, Santa Cruz, CA). Secondary antibodies were conjugated with horseradish peroxidase; blots were developed with an enhanced chemiluminescence (ECL) detection assay (Amersham Pharmacia Biotech, Piscataway, NJ). Membranes were then stripped and reprobed with antibodies to total JAK1, TYK2, STAT3 and GAPDH (to control for protein loading) and processed as described before. The intensity of the bands was analyzed by densitometry.

### Small Interference RNA (siRNA) Transfection by Electroporation

Fetal lung cells were transiently transfected using Nucleofector technology (Amaxa Biosystems). Freshly isolated E18 type II cells were plated on T75 flasks overnight. On the following day, cells were harvested by trypsinization, and aliquots of 2×10^6^ cells in RPMI with 10% FBS were centrifuged at 100 g for 10 min; supernatants were discarded and cell pellets were resuspended in 100 µl of basic Nucleofector solution (Primary Mammalian Epithelial Cell Protocol, Amaxa). Samples were mixed individually with different concentrations (0.5–4 µM) of siRNA SOCS3 (Thermo Scientific), or directed against the positive control GAPDH (Ambion, Applied Biosystems), transferred into the appropriate cuvettes and subjected to electrical pulses using the Nucleofactor II apparatus (Amaxa Biosystems). Samples containing no siRNA were otherwise treated in an identical manner and served as negative controls (pulse only). On the basis of previous studies from our laboratory [17], a T-13 program from Amaxa Biosystems was selected. After electroporation, samples were immediately transferred into Eppendorf tubes containing prewarmed RPMI plus 10% FBS and incubated at 37°C for 10 min. Cells were then transferred into Bioflex plates precoated with fibronectin and left undisturbed for 48 hours in a culture incubator. Following the 48 hours, monolayers were exposed to mechanical strain for 24 hours. Control conditions for each experiment included no transfection, eclectrical pulse only, nontargeted siRNA (negative control) and GAPDH siRNA (positive control). Each set of samples was subjected to Western blot analysis to confirm that SOCS3 was inhibited by siRNA.

### Adenoviral Infection

The premade recombinant adenoviruses encoding human wild-type caveolin-1 and caveolin-2 genes were obtained from commercial source (Capital Biosciences, Inc). Adenovirus encoding GFP (a kind gift from Dr. Jisu Li, Brown University) was used as negative control. Adenoviruses were amplified in HEK 293 cells, harvested, and purified with the Adeno-X Virus Purification Kit (Clontech, Mountainview, CA) according to the manufacturer’s recommendations. Adenoviral titers were determined using the Adeno-X Rapid Titer Kit (Clontech, Mountainview, CA) and expressed as multiplicity of infection (MOI). Subconfluent E18 monolayers were infected with adenoviruses at different MOI concentrations in medium containing serum-free DMEM plus 1.2 mM EGTA, pH = 7.4. After incubation for 90 min at 37°C, the virus-containing medium was removed and fresh serum-free DMEM was added.

### Statistical Analysis

Results are expressed as means ±SEM from at least 3 experiments, using different litters for each experiment. Data were analyzed with ANOVA followed by post hoc tests, and Instat 3.0 (GraphPad Software, San Diego, CA) was used for statistical analysis; *P*<0.05 was considered statistically significant.

## Results

### IL-10 Receptor and SOCS3 are Developmentally Regulated

**Figure 1:**
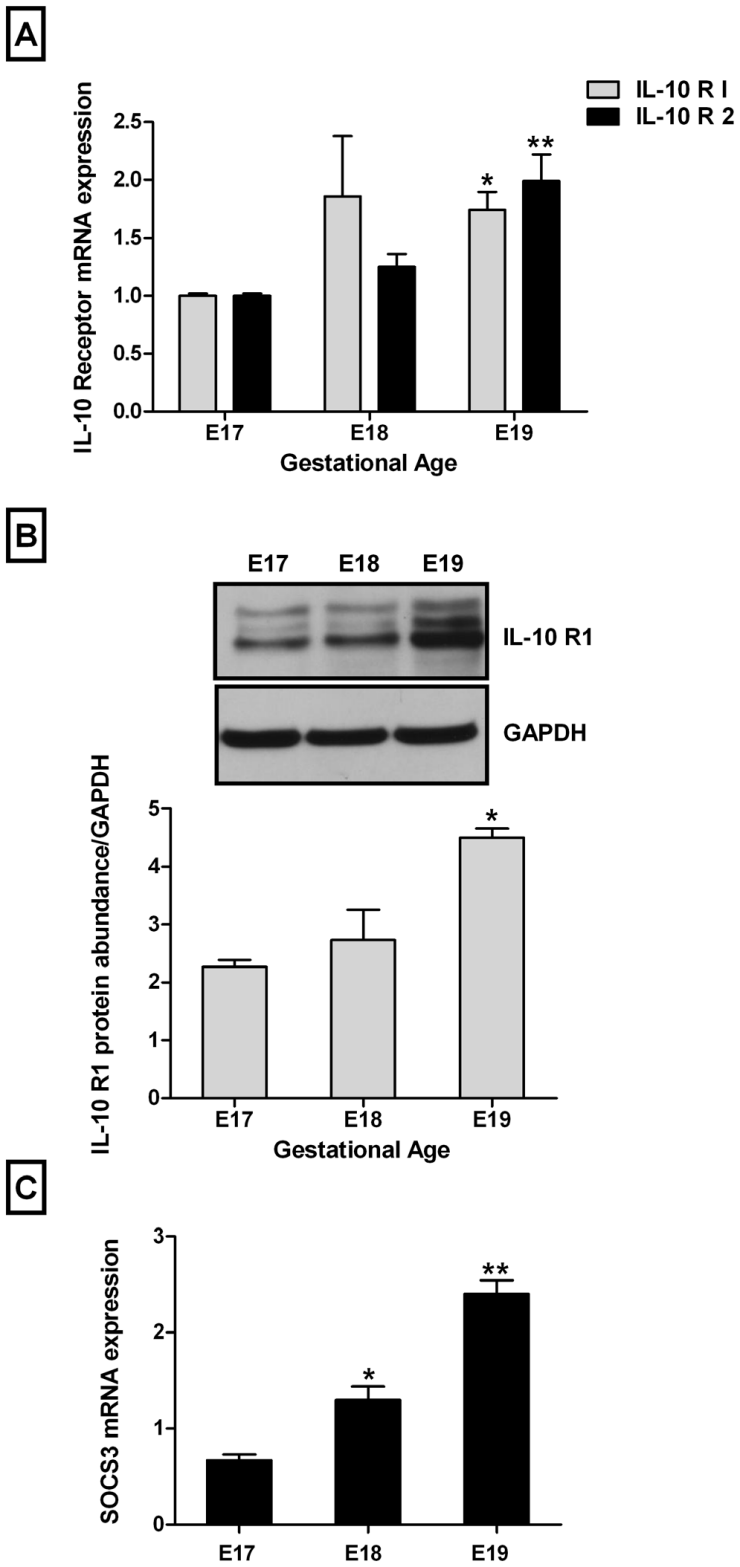
IL-10 receptor and SOCS3 expression levels increase with gestational age. Fetal type II cells were isolated at different gestational ages (E17–E19). Samples were processed and analyzed by real-time PCR for IL-10 receptor mRNA expression (**A**), by Western blot for IL-10 receptor protein abundance (**B**) and by real-time PCR for SOCS3 gene expression (**C**). *Upper panels* in **B** are representative blots. (*P<0.05 and **P<0.05 versus E17).

Previous experiments have shown that IL-10 mRNA expression in the bronchoalveolar lavage of newborns is inversely proportional to gestational age, suggesting that IL-10 expression may be developmentally regulated [18]. Therefore, we first looked at IL-10 receptor expression at various times during development. We investigated E17–E19 type II epithelial cells and corresponding IL-10 receptor expression for both IL-10 R1 and IL-10 R2. We found that both IL-10 R1 and IL-10 R2 gene expression increased with gestational age by 1.7-fold and 2-fold, respectively, at E19 when compared to E17 (1±0.02 vs. 1.74±0.16 and 1.99±0.23) (**Figure 1A**). Similar results were observed on IL-10 R1 protein abundance (2.27±0.12 vs. 2.73±0.52 and 4.50±0.16) (**Figure 1B**). SOCS3 mRNA expression also increased with advanced gestation (2-fold and 3.5-fold at E18 and E19, respectively when compared to E17) (0.67±0.06 vs. 1.30±0.14 and 2.40±0.14) (**Figure 1C**).

### Mechanical Stretch Decreases IL-10 Receptor and IL-10 Gene Expression and Increases SOCS3

**Figure 2:**
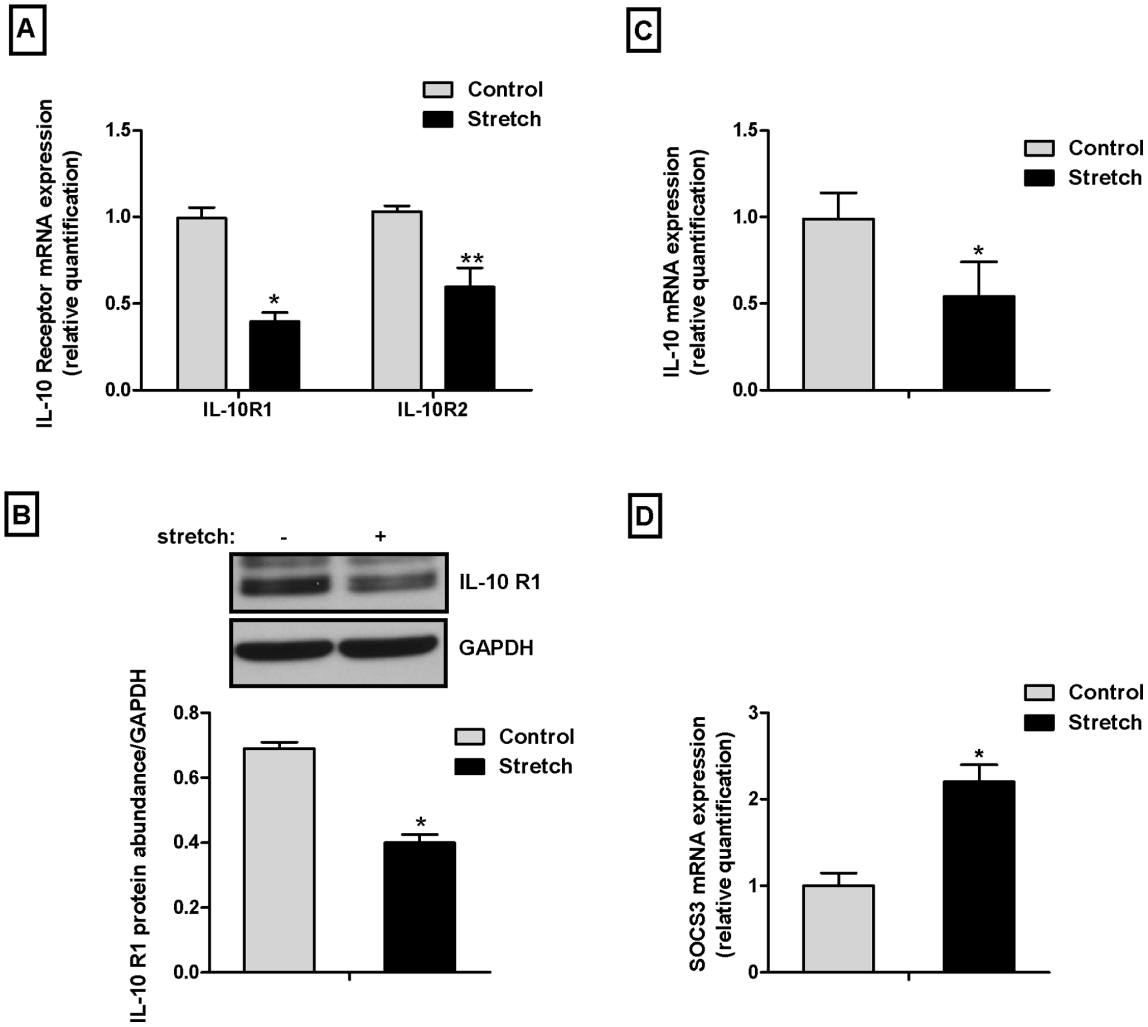
Mechanical stretch decreases IL-10 receptor and IL-10 gene expression and increases SOCS3. E18 type II epithelial cells were exposed to 20% cyclic stretch for 24 hours. (**A**) Samples were analyzed by real-time PCR for IL-10 receptor mRNA expression (n = 5, *P<0.0001, **P<0.0001 vs their respective controls). (**B**) By Western blot for IL-10 R1 protein abundance (n = 4, *P<0.05 vs control). (**C**) By real-time PCR for IL-10 gene expression (n = 4, *P<0.05). (**D**) By real-time PCR for SOCS3 gene expression (n = 3, *P<0.05).

Next, we studied the effect of mechanical stretch on IL-10 receptor and IL-10 gene expression. E18 type II epithelial cells were exposed to 20% cyclic stretch for 24 h and both IL-10 R1 and IL-10 R2 gene expression were analyzed as described in Methods. Following mechanical stretch, IL-10 R1 expression decreased by 60% and IL-10 R2 expression decreased by 42%, as compared to controls (0.99±0.06 vs. 0.40±0.05 and 1±0.03 vs. 0.59±0.11) (**Figure 2A**). IL-10 R1 protein also decreased by 58% compared to unstretched samples (0.69±0.02 vs. 0.40±0.02) (**Figure 2B**). Likewise, mechanical stretch decreased IL-10 gene by 50% when compared to controls (0.99±0.15 vs. 0.54±0.20) (**Figure 2C**). In contrast, mechanical stretch increased SOCS3 gene expression by 2-fold when compared to unstretched samples (1±0.15 vs. 2.2±0.20) (**Figure 2D**).

### Effect of Mechanical Stretch on IL-10 and IL-6 Secretion

**Figure 3:**
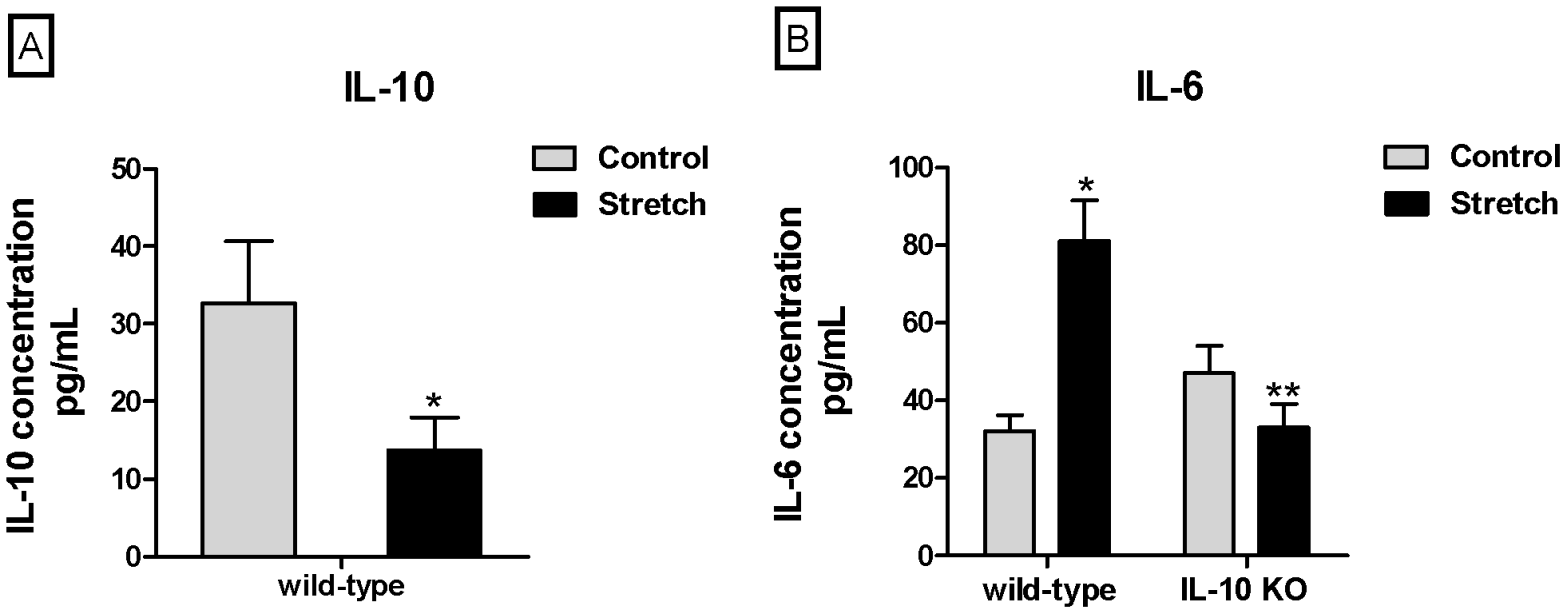
Effect of mechanical stretch on IL-10 and IL-6 release into the supernatant of type II epithelial cells. **A)** E18 type II cells were exposed to 20% cyclic stretch for 24 hours. Supernatant was collected and measured for concentrations of IL-10 by ELISA, as described in methods. Results were normalized to the cell lysate concentration in each sample (n = 5, *P<0.02 vs control). **B)** Samples were processed as above, except that IL-6 release was also measured in type II cells isolated from IL-10 knockout mice (n = 3, *P<0.05 vs control wild-type; n = 3, **P<0.05 vs control IL-10 KO).

Prior studies from our laboratory have shown that rat type II epithelial cells exposed to 20% mechanical stretch for 24 h decreased IL-10 secretion [7]. In order to determine whether mouse type II epithelial cells would respond in a similar fashion, we exposed E18 mouse type II epithelial cells to the same experimental conditions and examined the concentrations of IL-10 in the supernatant. We found that mechanical stretch decreased IL-10 concentration in the supernatant by 58% as compared to controls (32.6±8 vs. 13.7±4.3) (**Figure 3A**). In contrast, mechanical stretch increased release of the pro-inflammatory cytokine IL-6 by 2.3-fold as compared to unstretched samples (32±4.2 vs. 81±10.5) (**Figure 3B**). Interestingly, in cells isolated from IL-10 knockout mice, mechanical stretch decreased IL-6 secretion by 30% compared to controls (47±7 vs. 33±6).

### Effect of Mechanical Stretch on IL-10 Signaling Proteins in Wild-type Cells

**Figure 4:**
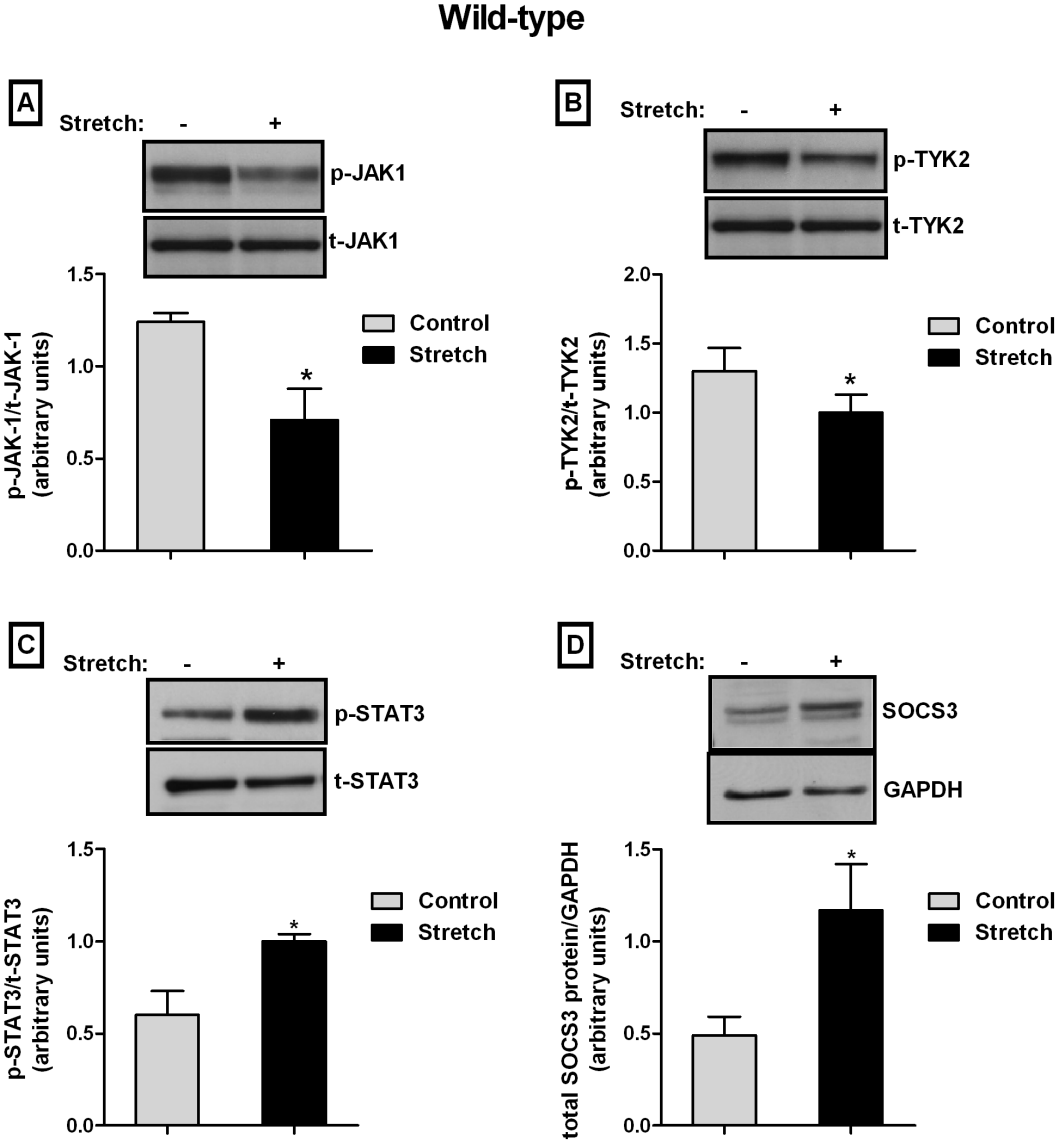
Effect of mechanical stretch on IL-10 signaling proteins in wild-type cells. E18 type II epithelial cells were exposed to 20% cyclic stretch for 24 h. Samples were analyzed by Western blot using phospho-JAK1 (**A**), phospho-TYK2 (**B**), phospho-STAT3 (**C**) and total SOCS3 (**D**) antibodies. Blots were reprobed with total antibodies and GAPDH (for SOCS3) to control for protein loading. *Top panels* are representative Western blots. Results are from 4 independent experiments. (*P<0.05 vs controls).

The binding of IL-10 to the receptor complex activates JAK1 (associated with IL-10 R1) and TYK2 (associated with IL-10 R2) which then enables further phosphorylation of the cytoplasmic tail of the receptor complex [8]. Given that IL-10 secretion is decreased following mechanical stretch, we investigated the effect of mechanical stretch on IL-10 pathway. Type II epithelial cells exposed to 20% cyclic stretch for 24 h showed a decrease in JAK1 phosphorylation by 43% when compared to controls (1.24±0.05 vs 0.71±0.17) (**Figure 4A**). Similarly, mechanical stretch for 24 h decreased TYK2 phosphorylation by 23% as compared to controls (1.30±0.17 vs 1±0.13) (**Figure 4B**). STAT3 phosphorylation is responsible for the transcription of IL-10 responsive genes [9], [19]. Therefore, we analyzed next the effect of mechanical stretch on STAT3 activation. Surprisingly, mechanical stretch for 24 h increased STAT3 phosphorylation by 1.7-fold as compared to controls (0.6±0.13 vs. 1±0.04) (**Figure 4C**). SOCS3 is one of the IL-10 responsive genes [10] and a negative feedback regulator of IL-10/TYK2/STAT pathway [20], [21]. Therefore, to assess whether SOCS3 could play any role in the decreased amount of IL-10 after mechanical stretch, we investigated the effect of mechanical stretch on SOCS3 expression level. Similar to STAT3 activation, our studies showed that type II epithelial cells exposed to mechanical stretch for 24 h increased SOCS3 protein abundance by 2.4-fold as compared to controls (0.49±0.1 vs. 1.17±0.25) (**Figure 4D**).

### Effect of Mechanical Stretch on IL-10 Signaling Pathway in Cells Derived from IL-10 Null Mice

**Figure 5:**
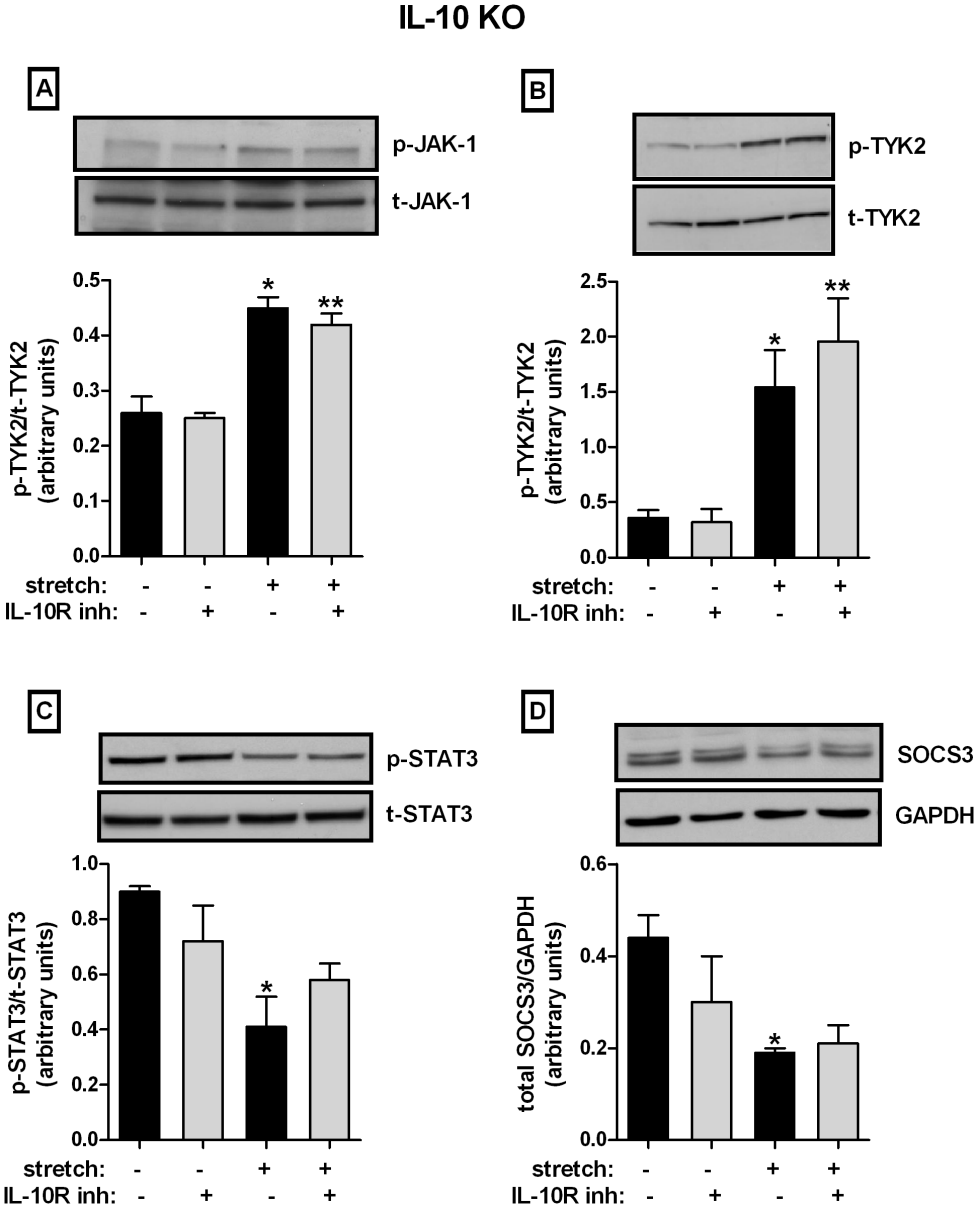
Effect of mechanical stretch on IL-10 signaling proteins in type II cells isolated from IL-10 knockout mice. Samples were subjected to the same experimental conditions as described in Figure 4, except that E18 type II epithelial cells were isolated from IL-10 knockout mice and some samples were preincubated with anti-IL-10 receptor antibody. *Top panels* are representative Western blots. Results are from 4 independent experiments. (*P<0.05 and **P<0.05 vs their respective controls).

Given the unexpected results of activation of some of the proteins of IL-10 signaling pathway by mechanical stretch and the interdependence between IL-10 and IL-6 secretions (figure 3B), we investigated the effect of mechanical stretch on IL-10 signaling pathway using cells isolated from IL-10 knockout mice. In addition, to assess the role of IL-10 receptor in the transduction of mechanical signal, IL-10 receptor was blocked using a functional antibody [22]. Mechanical stretch for 24 h in type II cells isolated from IL-10 knockout mice increased JAK-1 phosphorylation by 2.4-fold as compared to unstretched samples (0.26±0.03 vs. 0.45±0.02). Similar results were observed in cells incubated with anti-IL-10 receptor antibody (0.25±0.01 vs. 0.42±0.02) (**Figure 5A**). TYK2 was also activated by 4 to 6-fold after 24 h of stretch in the absence (0.36±0.07 vs. 1.54±0.34) or presence of neutralizing antibody (0.32±0.12 vs. 1.96±0.39) (**Figure 5B**). In contrast, mechanical stretch decreased STAT3 phosphorylation by 50% and 20% in the absence or presence of neutralizing antibody, respectively (0.90±0.02 vs. 0.41±0.11 and 0.72±0.13 vs. 0.58±0.06) (**Figure 5C**). Similarly, after 24 h of cyclic stretch SOCS3 protein decreased by 57% and 30%, respectively (0.44±0.05 vs. 0.19±0.01 and 0.30±0.1 vs. 0.21±0.04) (**Figure 5D**). All together, our data show an opposite effect of mechanical stretch on signaling proteins in type II cells isolated from IL-10 knockout mice compared to wild-type cells. In addition, the IL-10 receptor does not seem to play any significant role in the mechanotransduction of downstream signaling proteins.

### SOCS3 Decreases IL-10 Secretion and Increases IL-6 Release in Fetal Type II Cells Exposed to Mechanical Stretch

**Figure 6:**
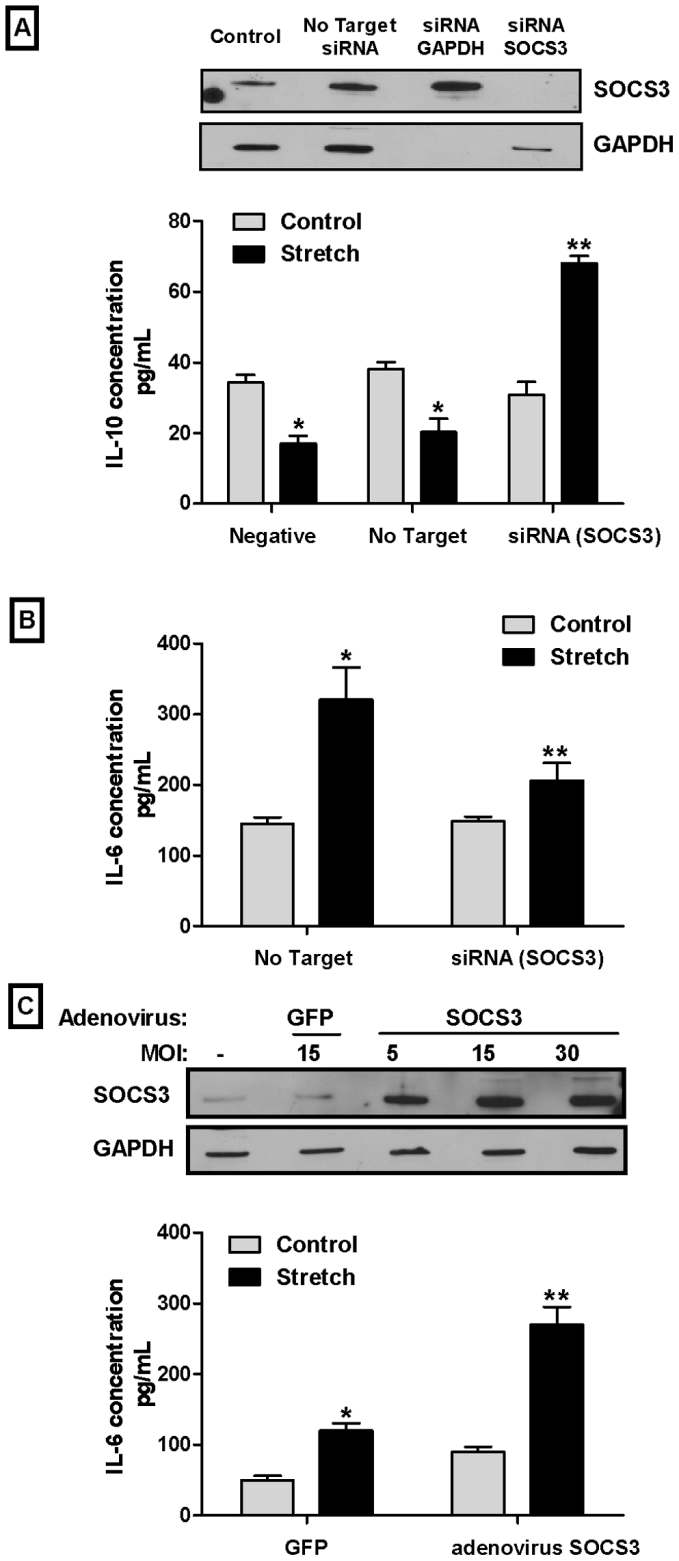
SOCS3 decreases IL-10 secretion and increases IL-6 release in fetal type II cells exposed to mechanical stretch. **A)** E18 type II epithelial cells were transfected with nothing (negative), no target or siRNA for SOCS3 [1 µM] and exposed to 20% mechanical stretch for 24 h. Supernatant was collected and IL10 concentration was determined by ELISA (n = 5, *P<0.05 vs their respective controls). The *upper panel* shows a representative Western blot demonstrating efficient inhibition of SOCS3 protein in samples treated with siRNA SOCS3 at 1 µM. **B)** E18 type II cells were processed as described in **A** and exposed to mechanical stretch for 24 h to measure release of IL-6 by ELISA (n = 4, *P<0.05 vs control; **P<0.05 vs stretch no target). **C)** E18 type II cells were infected with adenovirus expressing SOCS3 at multiplicity of infection (MOI) of 15 or negative control green fluorescent protein (GFP) at the same MOI. Two days later, monolayers were exposed to 20% stretch for 24 h. Supernatants were collected and IL-6 concentration was quantified by ELISA (n = 4, *P<0.05 vs control; **P<0.05 vs stretch GFP). The *upper panel* shows a representative Western blot showing increased expression of SOCS3 protein in samples infected with adenovirus encoding SOCS3.

To investigate further the role of SOCS3 in the decreased IL-10 secretion after mechanical stimulation, SOCS3 was knocked down by siRNA. Our data showed that IL-10 concentration in the supernatant of stretched samples without siRNA for SOCS3 or non-target siRNA decreased by 50% as compared to controls (34.3±2.17 vs. 17±2.31). In contrast, in samples transfected with siRNA for SOCS3, IL-10 release increased by 2-fold in stretched samples as compared to controls (30.8±3.8 vs. 68.1±2.1) (**Figure 6A**). These data strongly suggest that SOCS3 plays a key role in the decreased IL-10 secretion of type II cells after exposure to injurious stretch. To analyze whether the effect of SOCS3 on IL-10 signaling is direct or via IL-6 pathway, we next studied IL-6 secretion in SOCS3 knockdown cells exposed to mechanical stretch. As seen in **Figure 6B**, in non-target cells mechanical stretch increased IL-6 release by 2.2-fold (145±9.3 vs. 320±46). In contrast, in cells in which SOCS3 was knock-downed, mechanical stretch increased IL-6 release by only 1.4-fold (149±5.7 vs. 206±25), a 36% reduction when compared to stretched samples without siRNA SOCS3. Furthermore, in samples infected with adenovirus encoding SOCS3, mechanical stretch further increased IL-6 secretion by 2-fold when compared to stretched samples of adenovirus expressing GFP (120±10 vs. 270±25) (**Figure 6C**). Taken together these results show that SOCS3 inhibits IL-10 release and stimulates release of IL-6 in fetal type II cells exposed to mechanical stretch.

## Discussion

The cell signaling mechanisms responsible for the decrease of the anti-inflammatory cytokine IL-10 in fetal type II cells exposed to mechanical stretch are not known. Here we show that mechanical stretch of murine type II epithelial cells decreases IL-10, IL-10 receptors and their associated proteins JAK1 and TYK2 and increases IL-6, STAT3 and SOCS3. An opposite effect was observed in mechanically stretched type II cells isolated from IL-10 knockout mice. Interestingly, down-regulation of SOCS3 increased IL-10 secretion and decreased IL-6 secretion following mechanical stretch. These studies suggest that the inhibitory effect of mechanical stretch on IL-10 secretion is mediated via activation of IL-6-STAT3-SOCS3 signaling pathway.

Little is known about IL-10 receptor gene expression in type II epithelial cells and its relationship to gestational age. Our data show that IL-10 receptor gene expression increases with gestational age. These results are supported by prior studies demonstrating an inverse relationship between gestation age and IL-10 concentrations in the bronchoalveolar lavage of newborns [23]. Given that there is little difference in the ability to produce pro-inflammatory cytokines between preterm and term infants [24], decreased IL-10 receptors in premature infants may affect the ability to produce adequate amount of IL-10 in response to an inflammatory insult, such as mechanical ventilation. This hypothesis is further supported by our data showing that type II epithelial cells exposed to mechanical stretch resulted in the downregulation of the IL-10 receptor and IL-10 gene as compared to controls. Therefore, the combination of prematurity and mechanical stretch may have a significant impact in the ability to produce IL-10 to counterbalance and modulate an inflammatory response. The mechanisms by which mechanical stretch down-regulates IL-10 receptor are not fully understood. However, previous studies have shown that IL-10 receptor gene expression can be modulated by inflammatory cytokines [25].

IL-6 and IL-8 are thought to play key roles in the initiation of the inflammatory cascade in premature infants exposed to mechanical ventilation. Increased levels of these cytokines are found prior to the influx of neutrophils in the lung of preterm infants who develop bronchopulmonary dysplasia [26]. In addition, decreased concentrations of IL-10 have been observed in cultured human leukocytes from premature infants as compared to adults [27] and in preterm infants exposed to prolonged mechanical ventilation [28]. Our in vitro data are supported by these investigations and show that injurious stretch to fetal type II cells decreases the level of the anti-inflammatory cytokine IL-10 and increases release of the pro-inflammatory cytokine IL-6, showing. The overwhelming inflammatory response seen in mechanical ventilation, combined with the inability of type II epithelial cells to produce adequate IL-10 in response to mechanical stretch may be a critical component for the development of chronic lung disease.

The mechanisms underlying the decreased production of IL-10 in type II epithelial cell exposed to mechanical stretch remain unknown. We therefore investigated the effect of mechanical stretch on proteins from the IL-10 signaling pathway including JAK1, TYK2, STAT3 and SOCS3. JAK1 and TYK2 participate in the enzymatic activation and downstream recruitment of signaling proteins present in the IL-10 pathway [29]. Mice deficient in TYK2 for example have been shown to be susceptible to overwhelming viral and bacterial infections [30]. This supports the important presence of this kinase in regulating a functional immune response. Our data show that type II epithelial cells exposed to mechanical stretch had decreased activity of JAK1 and TYK2 as compared to controls. These findings support our hypothesis and suggest that inhibition of the IL-10 signaling pathway by stretch may contribute to the decreased production/release of IL-10. However, we did not anticipate that mechanical stretch activated STAT3 and SOCS3. These results can be interpreted in the context that these two proteins are not exclusive of the IL-10 signaling pathway and their activation can also be mediated via other cytokine receptors. STAT3 for example is an important transcription factor for the production of pro-inflammatory cytokines, such as IL-6 [31]–[32]. Expression of SOCS3 is also induced by numerous factors including Toll-like receptor agonists, IL-10, IL-6 and other gp130 signaling cytokines, leptin and IFN-γ [12], [33]. Previous studies from our laboratory [34] and current investigations (figure 3B) demonstrate that mechanical stretch of fetal type II cells increases release of IL-6. Therefore, stretch-induced increase of STAT3 and SOCS3 could be mediated via IL-6 pathway. Interestingly, when similar set of experiments were done in cells isolated from IL-10 knockout mice, mechanical stretch increased JAK1 and TYK2 phosphorylation and decreased STAT3 and SOCS3 stimulation (Figure 5). These results are completely opposite to the findings in wild-type cells. Given that we hypothesized that the increase of STAT3 and SOCS3 by stretch in normal cells was mediated by IL-6, we investigated IL-6 release in IL-10 knockout cells exposed to mechanical stretch. We observed that in the absence of IL-10, mechanical stretch decreased release of IL-6 (Figure 3B). These findings support our hypothesis that activation of STAT3 and SOCS3 by stretch is mediated via IL-6. However, these results are in apparent contradiction with previous studies showing that stimulation of LPS for example to cells isolated from IL-10 knockout mice increases release of IL-6 [35]. A potential explanation for these results is that in the absence of IL-10, mechanical stretch activates different signaling pathways and therefore activation of transcription factors leading to IL-6 expression may be defective.

To further investigate whether SOCS3 is a critical regulator of IL-10 secretion in fetal type II cells exposed to injurious stretch, SOCS3 gene was knocked down using siRNA. Following transfection with siRNA for SOCS3, type II cells exposed to mechanical stretch had an increased release of IL-10 as compared to controls. These results strongly suggest that SOCS3 plays a critical role in the decreased secretion of IL-10 seen in type II cells following mechanical stretch. The effect of SOCS3 deficiency or overexpression on IL-6-mediated inflammatory responses is still controversial. Using gain-and-loss of function approaches, our studies show that SOCS3 stimulates IL-6 secretion in fetal type II cells exposed to mechanical stretch given that in the presence of siRNA SOCS3 IL-6 secretion is decreased when compared to non-target. Likewise overexpression of SOCS3 using adenovirus vector further increased release of IL-6 when compared to control virus. From these studies we can conclude that in the presence of mechanical stretch, SOCS3 activates IL-6 and inhibits IL-10. In addition, these data show an interdependent and opposite relationship between IL-10 and IL-6 levels in fetal type II cells exposed to mechanical stretch.

The mechanisms by which inhibition of SOCS3 increases IL-10 are not clear. Forced expression of SOCS3 significantly suppressed the ability of IL-10 to trigger tyrosine phosphorylation of STAT3, suggesting that SOCS3 functions as a negative feedback regulator of IL-10/STAT3 signaling [20]. Therefore, decreased IL-10 production after mechanical stretch could be attributed to an increase of SOCS3 via IL-6 pathway; SOCS3 will then inhibit IL-10 receptor and signaling proteins. In addition, we cannot rule out the possibility that mechanical stretch could have a direct inhibitory effect on IL-10 receptors. However, blockade of this receptor with neutralizing antibody did not change the pattern of downstream signaling activation when compared to samples without receptor inhibitor, making this possibility less likely.

In summary, our findings show that activation of SOCS3 via IL-6 pathway plays a key role in the decrease of IL-10 secretion in fetal type II cells exposed to mechanical stretch. Inhibition of SOCS3 not only restores IL-10 secretion but also decreases IL-6 release in fetal type II cells exposed to mechanical stretch. The mechanisms by which SOCS3 regulates IL-10 and IL-6 secretion in the context of mechanical injury will require further investigations. Although in vivo models are needed to support our data, our in vitro studies suggest that SOCS3 may be a key potential target to minimize lung injury associated with mechanical ventilation in premature infants.

## References

[1] Baraldi E, Filippone M (2007) Chronic lung disease after premature birth. N Engl J Med 357: 1946–1955. 17989387 10.1056/NEJMra067279

[2] Fanaroff AA, Stoll BJ, Wright LL, Carlo WA, Ehrenkranz RA, et al.. (2007) Trends in neonatal morbidity and mortality for very low birthweight infants. Am J Obstet Gynecol 196: 147 e141–148. 10.1016/j.ajog.2006.09.014 17306659

[3] Bhandari A, Bhandari V (2003) Pathogenesis, pathology and pathophysiology of pulmonary sequelae of bronchopulmonary dysplasia in premature infants. Front Biosci 8: e370–380. 12700058 10.2741/1060

[4] Garingo A, Tesoriero L, Cayabyab R, Durand M, Blahnik M, et al (2007) Constitutive IL-10 expression by lung inflammatory cells and risk for bronchopulmonary dysplasia. Pediatr Res 61: 197–202. 17237722 10.1203/pdr.0b013e31802d8a1c

[5] McGowan EC, Kostadinov S, McLean K, Gotsch F, Venturini D, et al (2009) Placental IL-10 dysregulation and association with bronchopulmonary dysplasia risk. Pediatr Res 66: 455–460. 19581835 10.1203/PDR.0b013e3181b3b0fa PMC2795791

[6] Speer CP (2006) Inflammation and bronchopulmonary dysplasia: a continuing story. Semin Fetal Neonatal Med 11: 354–362. 16702036 10.1016/j.siny.2006.03.004

[7] Lee HS, Wang Y, Maciejewski BS, Esho K, Fulton C, et al (2008) Interleukin-10 protects cultured fetal rat type II epithelial cells from injury induced by mechanical stretch. Am J Physiol Lung Cell Mol Physiol 294: L225–232. 18065656 10.1152/ajplung.00370.2007

[8] Moore KW, de Waal Malefyt R, Coffman RL, O’Garra A (2001) Interleukin-10 and the interleukin-10 receptor. Annu Rev Immunol 19: 683–765. 11244051 10.1146/annurev.immunol.19.1.683

[9] Donnelly RP, Dickensheets H, Finbloom DS (1999) The interleukin-10 signal transduction pathway and regulation of gene expression in mononuclear phagocytes. J Interferon Cytokine Res 19: 563–573. 10433356 10.1089/107999099313695

[10] Mosser DM, Zhang X (2008) Interleukin-10: new perspectives on an old cytokine. Immunol Rev 226: 205–218. 19161426 10.1111/j.1600-065X.2008.00706.x PMC2724982

[11] Johnston JA, O’Shea JJ (2003) Matching SOCS with function. Nat Immunol 4: 507–509. 12774070 10.1038/ni0603-507

[12] Lang R, Pauleau AL, Parganas E, Takahashi Y, Mages J, et al (2003) SOCS3 regulates the plasticity of gp130 signaling. Nat Immunol 4: 546–550. 12754506 10.1038/ni932

[13] Dimitriou ID, Clemenza L, Scotter AJ, Chen G, Guerra FM, et al (2008) Putting out the fire: coordinated suppression of the innate and adaptive immune systems by SOCS1 and SOCS3 proteins. Immunol Rev 224: 265–283. 18759933 10.1111/j.1600-065X.2008.00659.x

[14] Wang Y, Maciejewski BS, Soto-Reyes D, Lee HS, Warburton D, et al (2009) Mechanical stretch promotes fetal type II epithelial cell differentiation via shedding of HB-EGF and TGF-alpha. J Physiol 587: 1739–1753. 19237431 10.1113/jphysiol.2008.163899 PMC2683961

[15] Tschumperlin DJ, Oswari J, Margulies AS (2000) Deformation-induced injury of alveolar epithelial cells. Effect of frequency, duration, and amplitude. Am J Respir Crit Care Med 162: 357–362. 10934053 10.1164/ajrccm.162.2.9807003

[16] Wang Y, Maciejewski BS, Drouillard D, Santos M, Hokenson MA, et al (2010) A role for caveolin-1 in mechanotransduction of fetal type II epithelial cells. Am J Physiol Lung Cell Mol Physiol 298: L775–783. 20172952 10.1152/ajplung.00327.2009 PMC2886604

[17] Wang Y, Maciejewski BS, Lee N, Silbert O, McKnight NL, et al (2006) Strain-induced fetal type II epithelial cell differentiation is mediated via cAMP-PKA-dependent signaling pathway. Am J Physiol Lung Cell Mol Physiol 291: L820–827. 16751225 10.1152/ajplung.00068.2006

[18] Jones CA, Cayabyab RG, Kwong KY, Stotts C, Wong B, et al (1996) Undetectable interleukin (IL)-10 and persistent IL-8 expression early in hyaline membrane disease: a possible developmental basis for the predisposition to chronic lung inflammation in preterm newborns. Pediatr Res 39: 966–975. 8725256 10.1203/00006450-199606000-00007 PMC7101752

[19] Donnelly RP, Sheikh F, Kotenko SV, Dickensheets H (2004) The expanded family of class II cytokines that share the IL-10 receptor-2 (IL-10R2) chain. J Leukoc Biol 76: 314–321. 15123776 10.1189/jlb.0204117

[20] Berlato C, Cassatella MA, Kinjyo I, Gatto L, Yoshimura A, et al (2002) Involvement of suppressor of cytokine signaling-3 as a mediator of the inhibitory effects of IL-10 on lipopolysaccharide-induced macrophage activation. J Immunol 168: 6404–6411. 12055259 10.4049/jimmunol.168.12.6404

[21] Sommer U, Schmid C, Sobota RM, Lehmann U, Stevenson NJ, et al (2005) Mechanisms of SOCS3 phosphorylation upon interleukin-6 stimulation. Contributions of Src- and receptor-tyrosine kinases. J Biol Chem 280: 31478–31488. 16000307 10.1074/jbc.M506008200

[22] Kullberg MC, Jankovic D, Gorelick PL, Caspar P, Letterio JJ, et al (2002) Bacteria-triggered CD4(+) T regulatory cells suppress Helicobacter hepaticus-induced colitis. J Exp Med 196: 505–515. 12186842 10.1084/jem.20020556 PMC2196050

[23] Oei J, Lui K, Wang H, Henry R (2002) Decreased interleukin-10 in tracheal aspirates from preterm infants developing chronic lung disease. Acta Paediatr 91: 1194–1199. 12463318 10.1111/j.1651-2227.2002.tb00128.x

[24] Blahnik MJ, Ramanathan R, Riley CR, Minoo P (2001) Lipopolysaccharide-induced tumor necrosis factor-alpha and IL-10 production by lung macrophages from preterm and term neonates. Pediatr Res 50: 726–731. 11726731 10.1203/00006450-200112000-00016

[25] Michel G, Mirmohammadsadegh A, Olasz E, Jarzebska-Deussen B, Muschen A, et al (1997) Demonstration and functional analysis of IL-10 receptors in human epidermal cells: decreased expression in psoriatic skin, down-modulation by IL-8, and up-regulation by an antipsoriatic glucocorticosteroid in normal cultured keratinocytes. J Immunol 159: 6291–6297. 9550434

[26] Munshi UK, Niu JO, Siddiq MM, Parton LA (1997) Elevation of interleukin-8 and interleukin-6 precedes the influx of neutrophils in tracheal aspirates from preterm infants who develop bronchopulmonary dysplasia. Pediatr Pulmonol 24: 331–336. 9407566 10.1002/(sici)1099-0496(199711)24:5<331::aid-ppul5>3.0.co;2-l

[27] Schultz C, Temming P, Bucsky P, Gopel W, Strunk T, et al (2004) Immature anti-inflammatory response in neonates. Clin Exp Immunol 135: 130–136. 14678274 10.1111/j.1365-2249.2004.02313.x PMC1808915

[28] Schultz C, Tautz J, Reiss I, Moller JC (2003) Prolonged mechanical ventilation induces pulmonary inflammation in preterm infants. Biol Neonate 84: 64–66. 12890939 10.1159/000071446

[29] Ghoreschi K, Laurence A, O’Shea JJ (2009) Janus kinases in immune cell signaling. Immunol Rev 228: 273–287. 19290934 10.1111/j.1600-065X.2008.00754.x PMC2782696

[30] Karaghiosoff M, Neubauer H, Lassnig C, Kovarik P, Schindler H, et al (2000) Partial impairment of cytokine responses in Tyk2-deficient mice. Immunity 13: 549–560. 11070173 10.1016/s1074-7613(00)00054-6

[31] Riley JK, Takeda K, Akira S, Schreiber RD (1999) Interleukin-10 receptor signaling through the JAK-STAT pathway. Requirement for two distinct receptor-derived signals for anti-inflammatory action. J Biol Chem 274: 16513–16521. 10347215 10.1074/jbc.274.23.16513

[32] Heinrich PC, Behrmann I, Muller-Newen G, Schaper F, Graeve L (1998) Interleukin-6-type cytokine signalling through the gp130/Jak/STAT pathway. Biochem J 334 (Pt 2): 297–314. 10.1042/bj3340297 PMC1219691 9716487

[33] Alexander WS, Hilton DJ (2004) The role of suppressors of cytokine signaling (SOCS) proteins in regulation of the immune response. Annu Rev Immunol 22: 503–529. 15032587 10.1146/annurev.immunol.22.091003.090312

[34] Hawwa RL, Hokenson MA, Wang Y, Huang Z, Sharma S, et al (2011) IL-10 inhibits inflammatory cytokines released by fetal mouse lung fibroblasts exposed to mechanical stretch. Pediatr Pulmonol 46: 640–649. 21337733 10.1002/ppul.21433 PMC3103753

[35] Saadane A, Soltys J, Berger M (2005) Role of IL-10 deficiency in excessive nuclear factor-kappaB activation and lung inflammation in cystic fibrosis transmembrane conductance regulator knockout mice. J Allergy Clin Immunol 115: 405–411. 15696103 10.1016/j.jaci.2004.10.044

